# Controlling for race/ethnicity: a comparison of California commercial health plans CAHPS scores to NCBD benchmarks

**DOI:** 10.1186/1475-9276-9-4

**Published:** 2010-01-25

**Authors:** John Zweifler, Susan Hughes, Rebeca A Lopez

**Affiliations:** 1 Department of Family and Community Medicine, Fresno Medical Education Program, University of California, San Francisco, 155 North Fresno Street, Fresno, CA, USA

## Abstract

**Background:**

Because California has higher managed care penetration and the race/ethnicity of Californians differs from the rest of the United States, we tested the hypothesis that California's lower health plan Consumer Assessment of Healthcare Providers and Systems (CAHPS^®^) survey results are attributable to the state's racial/ethnic composition.

**Methods:**

California CAHPS survey responses for commercial health plans were compared to national responses for five selected measures: three global ratings of doctor, health plan and health care, and two composite scores regarding doctor communication and staff courtesy, respect, and helpfulness. We used the 2005 National CAHPS 3.0 Benchmarking Database to assess patient experiences of care. Multiple stepwise logistic regression was used to see if patient experience ratings based on CAHPS responses in California commercial health plans differed from all other states combined.

**Results:**

CAHPS patient experience responses in California were not significantly different than the rest of the nation after adjusting for age, general health rating, individual health plan, education, time in health plan, race/ethnicity, and gender. Both California and national patient experience scores varied by race/ethnicity. In both California and the rest of the nation Blacks tended to be more satisfied, while Asians were less satisfied.

**Conclusions:**

California commercial health plan enrollees rate their experiences of care similarly to enrollees in the rest of the nation when seven different variables including race/ethnicity are considered. These findings support accounting for more than just age, gender and general health rating before comparing health plans from one state to another. Reporting on race/ethnicity disparities in member experiences of care could raise awareness and increase accountability for reducing these racial and ethnic disparities.

## Background

In 1995 the United States Agency for Healthcare Research and Quality launched what is now the Consumer Assessment of Healthcare Providers and Systems (CAHPS^®^) program in an effort to standardize health plan evaluation. The CAHPS Health Plan Survey, a voluntary survey designed to capture data on member experiences with care as a measure of health plan quality, is one of a family of CAHPS surveys designed for specific health care settings [1]. The CAHPS Health Plan Survey is incorporated into the National Committee for Quality Assurance (NCQA) accreditation process for health plans [2]. Consumer assessments are playing an increasing role as quality indicators and in enrollee guides [3]. U.S. News & World reports have ranked California commercial health plans lower than national counterparts in rankings based on performance on NCQA clinical quality measures for prevention and treatment, as well as CAHPS patient experience responses that are adjusted for variables including age, general health rating, and gender [4,5]. Only one California health plan was ranked in the top 25 percent of commercial health plans in their 2008 report. Even among California's highest ranked health plans, consumer assessment scores are consistently lower than prevention and treatment scores [4].

In 2007, California was home to 12 percent of the American population [6]. However, it accounts for one quarter of the total U.S. health-maintenance organization (HMO) enrollment [7]. HMOs can be broadly defined as health plans that offer prepaid, comprehensive health coverage for both hospital and physician services. Close to half of California's 35 million population is enrolled in an HMO [8]. Since higher HMO penetration in California may forecast higher HMO penetration in the rest of the nation, it is important to understand why member scores are lower in California.

One plausible explanation for lower HMO member ratings of care in California is that the racial and ethnic composition of California differs from the rest of the nation. It may be appropriate to consider race/ethnicity in member surveys because the Institute of Medicine includes "equity" as one of the six domains of health care quality [9]. CAHPS survey results are case-mix adjusted for age, gender, and self-reported health status, but although CAHPS surveys collect data on race/ethnicity, no adjustment is made for race/ethnicity [5]. Other research suggests individual health plans have a greater influence on member experiences of care than race/ethnicity, though this too is not a CAHPS-specific recommended adjustment [10,11].

Racial and ethnic disparities in survey responses are well documented. A 2003 study looking at Medicaid CAHPS data determined that consumer assessments of managed care varied by race, ethnicity and language, with racial/ethnic minorities reporting lower quality of care [12]. A study using the Medical Expenditure Panel Survey to assess enrollees in Medicaid managed care programs found Hispanics and Asian Americans reported less satisfaction with care than Whites [12]. Findings from the Commonwealth Fund 2001 Health Care Quality Survey showed only 56 percent of Hispanics and 45 percent of Asian Americans reported being "very satisfied" with their health care versus 65 percent of Whites [13]. Additionally, 15 percent of African Americans, 13 percent of Hispanics and 11 percent of Asian Americans felt they would have received better care if they were a different race or ethnicity.

A number of studies have shown that Asians and Pacific Islanders report different experiences with care than other races/ethnicities. Weech-Maldonado et al. reported Asian non-English speakers presented the lowest ratings of care of all the ethnicities and "had more negative reports and ratings of care" than Asian English speakers, whose ratings were similar to Whites [14]. Phillips et al. found Asian Americans the ethnic group most likely to report dissatisfaction with the quality of care provided by their usual source of care [12]. Taira et al. reported Asian Americans rated overall satisfaction, and 10 of 11 scales assessing primary care significantly lower than Whites [15]. They suggested that controlling for Asian-American ethnicity "could significantly improve the profiles of physicians with a large percentage of Asian-American patients." In a managed care setting, Asian patients as a group have been found to be "significantly less satisfied" with various aspects of primary care performance than other ethnicities [16].

California's diverse demographics may significantly impact its consumer assessment scores. Notably, there are more non-Whites than Whites in California with 35.9 percent Hispanic/Latino, 12.4 percent Asian, and 6.7 percent Black [17]. In 2007, California ranked 2^nd ^in the nation for percent of the total population who are Asian alone [18].

This study will test the hypothesis that California commercial health plan CAHPS survey results are attributable to the racial and ethnic composition of the state's population. California commercial health plan CAHPS survey responses will be compared to the 2005 National CAHPS Benchmark Database (NCBD) for selected measures. CAHPS survey results stratified by race/ethnicity will be presented and the impact of controlling for race/ethnicity as case-mix adjustments for California and nationally, will be discussed.

## Methods

National CAHPS 3.0 data for commercial adult health plan enrollees in the year 2005 was obtained from the National CAHPS Benchmarking Database (NCBD) after receiving approval from the UCSF Institutional Review Board (#06028447) and from NCBD. We did not analyze CAHPS data for Medicaid, State Children's Health Insurance Plan (SCHIP), or Medicare enrollees. Demographic characteristics measured included: age group, gender, race/ethnicity, insurance plan, type of insurance, time enrolled in insurance plan, educational level, general health rating, state of residence, report having a personal doctor, and report going to doctor's office or clinic in the last 12 months. These measures were selected to reflect the variables adjusted for in other similar surveys, and because in California they were found to be significantly different than the rest of the nation [19]. Nine questions from the CAHPS database were analyzed. Three questions were selected to reflect global ratings of doctor, health plan, and overall care. Six questions that comprise two composites were selected as indicators of interactions with providers that might be impacted by cultural competency and/or racial or ethnic differences: doctor communication and staff courtesy, respect and helpfulness. See Table 1 for a complete listing of the questions used. Two different scales were used to answer these questions: global ratings used a scale from 0 to 10, where 0 is the worst possible and 10 is the best possible; and all composite questions used a four point scale of never, sometimes, usually and always.

**Table 1:** Consumer assessments of healthcare providers and systems (CAHPS^®^) database questions and scales used

Global Rating Question			Scale										
What number would you use to rate your personal doctor or nurse?			0	1	2	3	4	5	6	7	8	9	10
			worst									best	
What number would you use to rate your health plan?			0	1	2	3	4	5	6	7	8	9	10
			worst									best	
What number would you use to rate all your health care in the last 12 months?			0	1	2	3	4	5	6	7	8	9	10
			worst									best	
**Composite: Staff Courtesy and Respect**			**Scale**										
In the last 12 months, how often did office staff at a doctor's office or clinic treat you with courtesy and respect?			Never		sometimes				usually			always	
In the last 12 months, how often were office staff at a doctor's office or clinic as helpful as you thought they should be?			Never		sometimes				usually			always	

**Composite: Doctor Communication**			**Scale**										
In the last 12 months, how often did doctors or other health providers listen carefully to you?			Never		sometimes				usually			always	
In the last 12 months, how often did doctors or other health providers explain things in a way you could understand?			Never		sometimes				usually			always	
In the last 12 months, how often did doctors or other health providers show respect for what you had to say?			Never		sometimes				usually			always	
In the last 12 months, how often did doctors or their health providers spend enough time with you?			Never		sometimes				usually			always	

We used the provided CAHPS categories for all of our demographic variables except race and insurance plan type. We combined the race and Hispanic variables in the database to incorporate Hispanic ethnicity into our race variable. Anyone who reported they were Hispanic in the Hispanic variable was coded as such regardless of race. Anyone reporting non-Hispanic in the Hispanic variable was coded as the race they specified in the race variable so our race categories were mutually exclusive. The type of insurance variable was collapsed into two categories: HMO and non-HMO. The non-HMO includes the CAHPS categories of POS, PPO, combined and other. Using the state of residence we created a variable to distinguish people in California from those in the rest of the nation (non-California).

In order to facilitate reporting and analysis, we chose to collapse each global rating score into two categories: highly rated or not highly rated. We followed the recommendation of Weech-Maldonado et al. (2008) and anyone responding either 9 or 10 on the worst to best scale was categorized as highly rated on that measure while all lower scores were categorized as not highly rated [20]. We followed CAHPS directions to create our composite variables [21]. We chose to equally weight the questions in each composite and added together all scores to get a possible total score of 16 for doctor communication (composite consists of four items) and 8 for staff courtesy, respect and helpfulness (composite consists of two items). We then collapsed these into highly rated categories based on scoring at least 90% of all possible points. Respondents scoring 14 or more on the doctor communication composite were categorized as highly rated. Those scoring 7 or 8 on the staff composite were categorized as highly rated. Scores lower than these were categorized as not highly rated.

We compared California to the rest of the nation using chi-square tests for the categorical demographic data. Chi-square tests were also used to look for differences in highly rated results for each global rating and both composite reports of care by race categories. We also compared California to the rest of the nation on each of these five measures using chi-square. Stepwise multiple logistic regression was used to see if ratings in California differed from all the other states combined (rest of the nation) on the five measures. Five regressions were done using the dependent measures of doctor rating, plan rating and care rating as well as reports on doctors communication and staff courtesy, respect and helpfulness. We used the following independent variables in each regression: age group (3 categories), education (6 categories), gender (2 categories), general health rating (5 categories), individual health plan (254 categories), insurance type (HMO or non-HMO), location (California or non-California), race/ethnicity (5 categories), and time in plan (4 categories). Fisher's exact tests were used to compare overall rankings of individual health plans for California to the rest of the nation using the logistic regression odds ratio results. Categories created based on the results of comparison to a New York HMO were: statistically higher, not statistically different, and statistically lower.

## Results

We analyzed 123,272 adult commercial health plan CAHPS respondents of which 9,952 were from California and 113,320 were from the rest of the nation. California adult commercial CAHPS participants differed from CAHPS participants in the rest of the nation in their demographic characteristics as well as their responses. Respondent characteristics are shown in Table 2. Californians are more likely to be 18-34 years old and less likely to be 65 years old or older. They are more likely to be males, more likely to have some college training, less likely to have more than a four year college degree, and more likely to be in an HMO. Californians are more likely than CAHPS participants in the rest of the nation to self identify as Hispanic or as Asian. They are also less likely to have a personal doctor or to have visited a doctor's office or clinic in the last 12 months.

**Table 2:** Respondent characteristics as defined and categorized by CAHPS^®^

Demographic	California n = 9,952	Rest of nation* n = 113,320
**Age (%)**		
18 - 34	16.8	15.9
35 - 64	73.5	71.4
≥ 65	8.5	12.2
Missing	1.3	0.5

Gender (%)		
Male	44.0	40.8
Missing	0.0	0.1

Race (%)		
White	54.3	77.0
Black	5.6	7.0
Asian/Pacific Islander	11.4	3.3
Hispanic	20.3	7.4
Other**	5.3	3.2
Missing	3.1	2.2

Insurance type (%)		
HMO	67.1	48.0
Non-HMO***	32.9	52.0

Time in plan (%)		
< 1 year	1.7	2.7
1 < 2 years	9.8	12.7
2 < 5 years	31.4	35.5
≥ 5 years	56.2	47.3
Missing	1.0	1.9

Education level (%)		
≤ 8^th ^grade	1.9	1.1
Some high school	3.8	3.2
High school graduate	21.7	24.9
Some college (AA)	36.4	32.9
College graduate (BA/BS)	17.2	18.2
More than 4 year degree	17.1	18.6
Missing	1.8	1.1

General Health Rating (%)		
Excellent	17.3	17.7
Very Good	41.0	40.0
Good	30.0	31.6
Fair	9.0	8.5
Poor	1.5	1.4
Missing	1.2	0.8

Do you have one person you think of as your personal doctor or nurse?		
No	22.6	11.2

In the last 12 months (not counting times you went to an emergency room), how many times did you go to a doctor's office or clinic to get care for yourself?		
None	24.1	14.4

* "Rest of nation" is defined as all states except California

**Includes American Indian, Other, and Multi-race

***non-HMO includes POS, PPO, Combined, and Other

Note: All categories were significantly different based on chi square test (p < 0.01)

Ratings in response to the five selected measures for California and the rest of the nation can be seen in Table 3. California CAHPS ratings were lower than the rest of the nation for each of the selected measures except health plan. A similar pattern was noted when we looked at racial and ethnic subgroups. In particular, Hispanics had the same response pattern with the exception that California health plans rated lower than national health plans, while Blacks in California rated their doctor, health plan, care, and staff courtesy, respect, and helpfulness higher, but their doctors communication lower in California than in the rest of the nation. Asians responded with the lowest scores on all measures in California as well as the rest of the nation.

**Table 3:** Percentage highly rated responses to selected CAHPS^® ^global ratings and composites for California and the nation

Rating/Report		White	Black	Asian/PI	Hispanic	Other	All
CA	Doctor *	51.4	61.9	43.9	53.5	52.8	51.3
	Plan *	43.7	53.3	38.9	47.2	39.5	43.9
	Care *	50.5	56.2	40.5	48.3	44.4	48.6
	Communication ^	64.0	67.6	55.6	64.4	59.4	62.9
	Staff ^	74.7	77.4	56.5	69.6	67.9	71.2

							

Nation	Doctor *	52.5	57.4	50.0	56.4	54.6	53.0
	Plan *	41.6	46.8	38.3	47.7	38.3	42.1
	Care *	54.5	54.8	45.9	54.4	48.9	53.9
	Communication ^	66.0	68.3	57.6	66.0	64.0	65.7
	Staff ^	76.1	75.8	63.7	72.2	72.6	72.2

* rating of 9 or 10

^ composite report score 90% of total possible

Note: All categories were significantly different based on chi square test (p < 0.01)

Stepwise logistic regression results agreed that the seven independent variables that contributed to the model in order of significance were: age, general health rating, education, individual health plan, time in plan, race/ethnicity, and gender. Variables that were not significant included type of insurance and California location. Table 4 shows the odds ratios with confidence intervals for all five rating/report characteristics and six of the seven significant independent variables. In the interest of space, the 254 individual health plans were not included in Table 4. In general, individuals with high ratings of their doctor, plan and care tended to be older, in better health, have less than a high school diploma, be in their plan longer, not be of Asian race, and be female. Composite reports of care regarding communication and staff courtesy differed from ratings by gender; males had higher rates than females. Reports of staff courtesy also differed by race/ethnicity. Only Blacks reported higher results, all other races were lower than Whites. In an overview of the individual health plans odds ratios, California plans did not differ from the rest of the nation except for the care rating where more California plans were statistically higher (data not shown).

**Table 4:** Odds ratio (95% confidence interval) results for 5 logistic regression models

	Model dependent variable - Global Ratings		
Independent variable	Doctor	Plan	Care
**Age**			
18 - 34 years	0.78 (0.76-0.81)	0.78 (0.76-0.81)	0.66 (0.64-0.69)
35 - 64 years^R^	1.00	1.00	1.00
≥ 65 years	1.56 (1.49-1.63)	2.63 (2.52-2.75)	1.81 (1.72-1.90)
**General Health Rating**			
Poor	0.85 (0.76-0.95)	0.72 (0.65-0.80)	0.49 (0.44-0.55)
Fair	0.75 (0.71-0.79)	0.66 (0.63-0.70)	0.53 (0.50-0.55)
Good	0.78 (0.76-0.80)	0.71 (0.68-0.73)	0.66 (0.64-0.68)
Very Good^R^	1.00	1.00	1.00
Excellent	1.47 (1.42-1.53)	1.57 (1.52-1.63)	1.64 (1.57-1.70)
**Education**			
< 8^th ^grade	1.09 (0.95-1.23)	1.32 (1.16-1.49)	1.09 (0.95-1.24)
Some high school	1.21 (1.12-1.31)	1.34 (1.24-1.44)	1.33 (1.22-1.44)
High school graduate^R^	1.00	1.00	1.00
Some college	0.88 (0.85-0.91)	0.74 (0.71-0.76)	0.85 (0.82-0.88)
BA/BS college degree	0.72 (0.69-0.75)	0.55 (0.53-0.57)	0.67 (0.64-0.70)
Graduate school	0.73 (0.70-0.76)	0.50 (0.48-0.52)	0.67 (0.64-0.70)
**Individual health plan**			
254 plans not included due to size constraints			
**Time in plan**			
< 1 year	0.80 (0.74-0.87)	0.65 (0.59-0.70)	0.78 (0.71-0.85)
1 to < 2 years	0.82 (0.79-0.86)	0.72 (0.69-0.75)	0.82 (0.78-0.85)
2 to < 5 years	0.83 (0.81-0.86)	0.78 (0.75-0.80)	0.84 (0.82-0.87)
≥ 5 years^R^	1.00	1.00	1.00
**Race/Ethnicity**			
Asian	0.88 (0.82-0.95)	0.96 (0.89-1.03)	0.77 (0.71-0.83)
Black	1.30 (1.23-1.37)	1.37 (1.30-1.44)	1.17 (1.11-1.24)
Hispanic	1.14 (1.08-1.21)	1.33 (1.26-1.39)	1.07 (1.01-1.13)
Other	1.15 (1.06-1.23)	0.96 (0.90-1.03)	0.90 (0.83-0.97)
White^R^	1.00	1.00	1.00
**Gender**			
Female	1.16 (1.13-1.19)	1.13 (1.10-1.16)	1.07 (1.04-1.10)
Male^R^	1.00	1.00	1.00

	**Model dependent variable - Composites**		
**Independent variable**	**Communication**	**Staff**	
**Age**			
18 - 34 years	0.75 (0.73-0.78)	0.69 (0.67-0.72)	
35 - 64 years^R^	1.00	1.00	
≥ 65 years	1.32 (1.25-1.38)	1.70 (1.61-1.81)	
**General Health Rating**			
Poor	0.40 (0.36-0.45)	0.53 (0.47-0.59)	
Fair	0.50 (0.47-0.52)	0.61 (0.58-0.65)	
Good	0.66 (0.64-0.68)	0.75 (0.73-0.78)	
Very Good^R^	1.00	1.00	
Excellent	1.64 (1.57-1.71)	1.38 (1.32-1.45)	
**Education**			
< 8^th ^grade	1.11 (0.97-1.28)	0.90 (0.77-1.05)	
Some high school	1.17 (1.07-1.27)	1.18 (1.07-1.30)	
High school graduate^R^	1.00	1.00	
Some college	0.85 (0.82-0.88)	0.81 (0.78-0.84)	
BA/BS college degree	0.73 (0.70-0.76)	0.69 (0.66-0.72)	
Graduate school	0.72 (0.69-0.75)	0.65 (0.62-0.68)	
**Individual health plan**			
254 plans not included due to size constraints			
**Time in plan**			
< 1 year	0.91 (0.83-1.00)	0.88 (0.80-0.97)	
1 to < 2 years	0.87 (0.84-0.91)	0.90 (0.85-0.94)	
2 to < 5 years	0.91 (0.88-0.94)	0.92 (0.89-0.95)	
≥ 5 years^R^	1.00	1.00	
**Race/Ethnicity**			
Asian	0.72 (0.66-0.78)	0.53 (0.49-0.58)	
Black	1.28 (1.21-1.36)	1.15 (1.09-1.23)	
Hispanic	1.05 (1.00-1.12)	0.90 (0.85-0.95)	
Other	0.97 (0.90-1.05)	0.87 (0.80-0.95)	
White^R^	1.00	1.00	
**Gender**			
Female	0.84 (0.82-0.87)	0.82 (0.79-0.84)	
Male^R^	1.00	1.00	

^R ^denotes the referent group

Note: All independent variables were significant with p < 0.001

## Discussion

We hypothesized that racial and ethnic differences in member experience of care scores, coupled with the higher concentration of Hispanics and Asian/Pacific Islanders in California would explain the lower scores California commercial health plans have received on CAHPS surveys. However, our analysis of adult enrollees in commercial health plans found that race/ethnicity was only one of multiple variables that influenced responses to selected CAHPS questions in California and the rest of the nation. When these variables were all accounted for, CAHPS scores for California health plans were no different than the rest of the nation. We found that the variables that contributed first in our stepwise logistic regression were age, general health rating, education, and health plan. Time in plan, race/ethnicity, and gender were also significant, but they were consistently less influential than the four variables noted above. Our findings suggest that national comparisons of patient experiences of care should adjust for a broader range of variables.

### Californians enrolled in commercial health plans

Although we found that California commercial health plan enrollee responses to selected questions in the CAHPS survey did not differ from the rest of the nation, California health plan enrollees did differ in several respects. First, there is a higher percentage of Californians enrolled in HMOs. Consequently, it is possible that Californians are more familiar with managed care and/or may have different expectations of HMO care than the rest of the nation. This familiarity and/or differing expectations could be reflected in CAHPS responses. Testing this hypothesis would require linking HMO penetration data not available in the CAHPS database, with CAHPS responses stratified by geography. It is possible that patient experience responses from commercial HMO enrollees differ from how enrollees in non-participating HMOs would respond. However, patient experience CAHPS surveys are mandated if an HMO seeks NCQA accreditation, and over 90% of all commercial HMO enrollees in California are in plans that have received NCQA accreditation [22].

California also differs from the rest of the nation in that a higher percentage of patients are cared for by large medical groups that contract with health plans to provide the majority of managed care services [23]. This "delegated model" transfers responsibility for many patient care decisions including authorization of diagnostic, inpatient, and specialty services from the health plan to the medical group. This may shield health plans from patient frustrations associated with accessing health care services while exposing the physicians that constitute the medical groups to more critical scrutiny and hence lower CAHPS scores. In unadjusted scores, Californians rated their health plan itself higher than enrollees around the nation rated their health plan. Conversely, California health plan enrollees rated their doctor, and their care, lower than health plan enrollees in the rest of the nation. However, these differences were not significant after controlling for other variables in our logistic regression. Furthermore, if lower patient reports of care were attributable to the delegated model, we would expect to see differences in CAHPS responses between Kaiser's group model and the other California commercial health plans that rely on the delegated model. In contrast, CAHPS scores for Kaiser, a group model HMO that serves close to one third of the commercial HMO members in California, are similar to CAHPS scores for other California commercial health plans [24,25].

We found that California commercial health plan enrollees rated their experiences of care similarly to commercial enrollees in the rest of the nation, but that may not mean the care itself is comparable. However, on a measure of clinical quality, the Health Plan Employer Data Information Set (HEDIS^®^), California commercial health plans scores are comparable to health plans across the nation [26]. Commercial enrollees in California differed from the rest of the nation in that Californians were less likely to have a personal doctor or to have visited a doctor's office or clinic in the last 12 months. This could suggest that Californians are healthier, have less access to care, or are otherwise incentivized to not have a personal doctor or seek care. It is unclear how these differences would impact our findings.

### Race/Ethnicity Differences

Our comparison of California to the rest of the nation's commercial CAHPS scores showed that race/ethnicity was only part of the reason scores differ. We found consistent variation in CAHPS scores between racial and ethnic groups. In both California and the rest of the nation, Blacks were significantly more likely to rate their doctor, their plan, and their care higher than Whites; Asians were more likely to rate their care, courtesy, understanding, and respect lower than Whites; and Hispanics were more likely to rate their plan higher than Whites.

One explanation for why racial and ethnic groups score their doctor, care, courtesy, understanding, and respect lower in California, as well as the nation as a whole, is discordance between physicians and patients. While California's population is very diverse, its physicians do not reflect the state's racial and ethnic distribution [27]. It is possible that CAHPS scores that assess interactions with their physician are lower if the physician is from a different racial or ethnic group. Some but not all studies suggest that patient-physician concordance impact patient ratings of care [28-30]. However, we are unable to investigate patient-physician concordance in the CAHPS findings because physician race/ethnicity is not collected.

It is also possible that the race/ethnicity of commercial health plan CAHPS respondents does not reflect the demographics of all commercial enrollees because certain racial/ethnic groups may be less comfortable responding to surveys than other groups. We are unable to account for this potential source of variation because we do not have racial or ethnic data for commercial health plan enrollees who do not respond to the CAHPS survey. We also cannot comment on whether the differences in CAHPS scores reflect the perceptions of all Californians in the different racial/ethnic groups, or only those Californians who are enrolled in commercial health plans.

Our analysis of commercial CAHPS scores does not explain why responses vary by race/ethnicity. If CAHPS responses vary by race/ethnicity because of differences in how CAHPS questions are interpreted rather than the care received, then not controlling for race/ethnicity would confound comparing member ratings of experiences of care in geographic regions with differing concentrations of Asians, Blacks and Hispanics. However, if health care does truly vary by race/ethnicity, then controlling for race/ethnicity would obscure real differences in care that would be important to track. It is of interest to note that another large scale member satisfaction survey in California, the Patient Assessment Survey sponsored by The California Cooperative Healthcare Reporting Initiative does control for race/ethnicity [31].

### Study Limitations

Our analysis has several limitations. Member participation in CAHPS surveys is voluntary, so participants may vary from all health plan enrollees in demographics and in perceptions of care. Most health plans are confined to individual states, limiting our ability to compare health plans across state lines [32]. CAHPS surveys already control for age, general health rating, and gender, but our analysis found that other variables including individual health plan, education, time in plan as well as race/ethnicity influenced responses to selected CAHPS questions. There may be other variables influencing CAHPS responses that we did not consider. For example, intrastate geographic differences in access to care and participation rates in CAHPS may account for some of the variation in responses.

Our analysis was limited to adult commercial enrollees. We did not look at health plan enrollees with other types of health insurance such as Medicare, Medicaid, or SCHIP, or at other age groups such as children. However, comparing experiences of care ratings across product lines would be difficult to interpret because of differences in reimbursement formulas and in enrollees.

### Measuring Equity

If race/ethnicity information was routinely collected by commercial health plans we could determine if CAHPS respondents differed from commercial health plan enrollees as a whole. Race/ethnicity data for commercial health plan enrollees would also allow us to look for disparities in other types of quality metrics such as HEDIS^®^. Regardless of whether the race/ethnicity differences in member scores are driven by cultural biases in survey responses or are due to real differences in care, our analysis supports the wider application of comparisons that consider race/ethnicity.

## Conclusion

California health plan enrollees rate their care similarly to health plan enrollees in the rest of the nation when we controlled for age, general health rating, education, individual plan, race/ethnicity, time in plan, and gender characteristics. Standardizing populations for these variables prior to comparing individual health plans or health plans by state would lessen the likelihood of ascribing differences in member reports of experiences of care to the health plan or state, when they may have more to do with the enrollees themselves.

Stratifying member experiences of care by race/ethnicity highlighted important differences in CAHPS ratings between different racial and ethnic groups in both California and the nation. Reporting on race/ethnicity disparities in member experiences of care could raise awareness and increase accountability for reducing racial and ethnic disparities, especially among individual health plans. Wider dissemination of public reporting which accounts for race/ethnicity and incentive programs that reward reducing disparities in care may help achieve these objectives.

## Competing interests

The authors declare that they have no competing interests.

## Authors' contributions

JZ conceived of and designed the study. SH participated in the design of the study and performed the statistical analysis. The first draft was written by JZ, SH, and RAL. Subsequent versions were revised by JZ, SH, and RAL; all authors read and approved the final manuscript.
